# Growing Up with Parental Mental Illness and Post-Traumatic Growth

**DOI:** 10.1007/s40653-025-00762-6

**Published:** 2025-09-13

**Authors:** Phoebe N. Hodgkins, Bahar Tunçgenç

**Affiliations:** 1 Department of Psychology, Nottingham Trent University, 50 Shakespeare Street, Nottingham, NG1 4FQ UK; 2 Institute of Human Sciences, The Pauling Centre, University of Oxford, 58a Banbury Rd, Park Town, Oxford, OX2 6QS UK

**Keywords:** Post-traumatic growth, Family, Childhood, Parental mental illness, Mental health

## Abstract

Globally, it is estimated that 4–23% of children have at least one parent who experiences a mental illness. Whilst the negative effects of growing up with a parent with a mental illness (PWMI) are well documented, potential positive effects such as building resilience, independence or empathy are often overlooked (Kinsella et al., 1996). Adopting a post-traumatic growth (PTG) framework, this study examined the positive effects of growing up with a PWMI. Adult participants who grew up with a PWMI (*N =* 71) and those who did not but still experienced a different type of highly stressful life event (*N* = 75) completed a survey about their parents’ mental health conditions, their own mental health status, and the Post-Traumatic Growth Index. Results showed participants who grew up with a PWMI had significantly higher levels of personal strengths compared to participants who did not. Among those who grew up with a PWMI, participants who did not have a mental illness themselves had significantly higher levels of personal strengths than those who had a mental illness. No other significant findings were found across the groups. This study provides proof-of-concept for the applicability of PTG theory to the largely overlooked, yet important area of mental health. The results suggest that growing up with PWMI may help build resilience through dealing with adult-like situations and effectively coping with adversities, provided that people have sufficient personal resources.

## Highlights

Whilst growing up with a parent with a mental illness (PWMI) has well-documented negative effects on an individual, it can, in theory, also lead to post-traumatic growth (PTG) through building resilience and independence.

This quantitative survey study reveals increased personal strengths in adults who had vs. who did not have a PWMI while growing up.

Among participants who grew up with a PWMI, those who did not have a mental illness themselves had significantly higher levels of personal strengths than those who had a mental illness themselves.

Findings reveal that individuals who grew up with a PWMI can build resilience through dealing with adult-like situations and effectively coping with adversities, provided that they have sufficient personal resources.

## Introduction

In the United Kingdom it has been estimated that 57% of men and 65% of women who have a mental illness are parents (Royal College of Psychiatrists, 2016). This equates to around 2.9 million children having a parent with a mental illness (PWMI) in England alone (Children’s Commissioner, 2019). Having a PWMI is considered an Adverse Childhood Experience, this equates to having experienced highly stressful events during childhood (Scott et al., 2021). These experiences can have harmful effects on the immunological, endocrine, and neurological development of children (Nelson et al., 2020).

The literature on growing up with a PWMI points to its numerous negative effects throughout the lifespan. These children often take on caring roles to support their parents (Dobener et al., 2022), which results in boundary distortions between the parent and child, and the child taking on developmentally distorted levels of responsibility such as preparing food and emotionally comforting their parent (Van Loon et al., 2017). Although engaging in caregiving can enhance resilience, maturity, and self-efficacy, these positive outcomes are dependent on the individual not having an inordinate number of responsibilities, and tasks not exceeding their maturity level (Borchet et al., 2021). If the caregiving roles are seen as overwhelming and a burden to the child, then this can result in them feeling depressed and anxious (Arellano et al., 2018). In fact it has been contested that if the roles of child and parent are reversed, also known as “parentification”, it can lead to the child experiencing deficits in their own health and their ability to form attachments (Hooper et al., 2008). Qualitative studies on adults who grew up with a PWMI also show social effects as these individuals struggle with trusting others, which may lead to relationship problems in adulthood (Källquist & Salzmann-Erikson, 2019). Furthermore, adults who grew up with a PWMI have been shown to have high externalising (e.g., defiant behaviours like aggression) and internalising issues (e.g., depression and anxiety) (Foster, 2010; Patrick et al., 2020), as well as an increased chance of developing a mental illness themselves (Herzog & Schmahl, 2018; Kinsella et al., 1996) However, it has long been recognised that positive perceptions can also be developed after experiencing adverse life events, including traumas (Tedeschi & Calhoun, 1996).

Although most of the literature reports negative effects of having a PWMI, some positive effects have been identified as well. For instance, adults with a PWMI often allude to gaining empathy, resilience, and patience (Metz & Jungbauer, 2021). Studies also suggest that adults who grew up with a PWMI experience a “steeling effect”, where they mature faster, their self-view positively changes and resilience grows as they successfully cope with their parent’s mental illness (Rutter, 2012; Van Parys et al., 2015). In the qualitative literature, it is common to see adults reflecting on their childhood highlighting positive aspects of having a PWMI and gratitude for the skills they developed (Metz & Jungbauer, 2021). Such reflection and meaning-making are key components of post-traumatic growth (PTG), which point to the potential for adults who grew up with a PWMI to develop PTG (Xu et al., 2016).

PTG is considered a positive psychological change caused from experiencing a highly stressful or traumatic event (Tedeschi & Calhoun, 1996). Following an event or series of events, it can cause an individual to positively restructure their values, cognitive processes, and priorities which can help them to appreciate their new sense of who they are (Muldoon et al., 2019). Positive changes can include stronger personal relationships, greater personal strength, and a sense of new possibilities, greater appreciation of life and a greater openness to spirituality (Tedeschi & Calhoun, 1996). PTG is not only a positive recovery following a traumatic event, but it is considered a transformative process during which individuals make meaning from their trauma, positively restructure their values, cognitive processes, and priorities (Muldoon et al., 2019), and ultimately reach a better level of functioning than what they had pre-trauma (Ogińska-Bulik & Kobylarczyk, 2016). However, traumatic experiences are not the direct cause of PTG. Instead, they can rather be seen as a catalyst to the individual engaging in meaning-making following the traumatic event (Xu et al., 2016). This is a vital step in changing previously held negative schemas which helps facilitate the development of PTG (Xu et al., 2016).

Experiencing PTG is linked to the individual’s views about themselves, others and the meaning of the event being positively changed, which allows for the development of positive coping strategies (Bianchini et al., 2017). Reflection and rumination about the traumatic event are considered key cognitive processes when developing PTG (Wu et al., 2015). Therefore, PTG is associated with being able to effectively cope in stressful situations and to self-reflect and develop a better mindset (Bryngeirsdottir & Halldorsdottir, 2021). However, PTG is also positively correlated with posttraumatic stress, meaning that it is not always associated with positive effects (Liu et al., 2017). PTG is widely researched in relation to single traumatic events, but a few studies have examined continuous or multiple traumas. It has been found that individuals who experienced a single traumatic event had higher levels of PTG compared to individuals who had continuous trauma such as child neglect and abuse, which can be applied to adults who grew up with a PWMI (Shuwiekh et al., 2018). However, a study examining adolescents who experienced multiple traumas during childhood found that trauma severity and post-traumatic stress symptoms were correlated with PTG (Fraus et al., 2021).

Limited in the literature is PTG in relation to mental illness – whether that be one’s own mental illness or growing up with a PWMI. The research that has been conducted typically examines PTG in individuals who have psychosis, as individuals who have a serious mental illness can experience high levels of trauma (Mazor et al., 2016). A traumatic experience is defined as an event that is, or perceived to be, life-threatening or physically damaging, involves extreme fear and feeling helpless (American Psychiatric Association, 2013). Having a PWMI can be deemed as traumatic as adults who grew up with a PWMI can experience events such as their parent attempting suicide or violent behaviour (Metz & Jungbauer, 2021). This could lead to the individual developing adverse behaviour patterns to help cope with stress, but could negatively affect them in the longer term, for example drug-use or isolation and even post-traumatic stress disorder (Center for Substance Abuse Treatment, 2014).

To our knowledge, there is only one quantitative study that has examined PTG in adults who grew up with a PWMI. Ergün et al. (2018) found that young adults who grew up with a PWMI did not experience PTG, but rather showed higher levels of resilience compared to young adults who did not grow up with a PWMI. However, the criteria only allowed participants whose parents were alive and still receiving treatment for their mental health. This limits the generalisability of the results as previous studies have highlighted that parents who have a mental illness do not always receive professional help as they often face obstacles such as lack of accessibility and the fear that social services may take their children away from them (Jones et al., 2016). Ergün et al. (2018) also used participants who were aged 18–23; however, PTG can occur weeks or years after a traumatic event, which could mean that young adults may have not had enough time to reflect and develop PTG (Chen et al., 2015). Lastly, participants who themselves had a mental illness were excluded, which is an important dimension to explore given the mixed evidence on whether people with a mental illness experience more, less, or similar amounts of PTG (Bianchini et al., 2017; Siqveland et al., 2015). Adopting this PTG approach to mental illnesses, this study will investigate whether growing up with a PWMI and having a mental illness can have positive effects for individuals.

To address these gaps, this study aims to address the paucity in research of the positive effects of experiencing parental mental illness by applying PTG theory and investigating the extent to which adults who grew up with a PWMI experienced PTG compared to adults who did not and by also considering the mental health status of those adults themselves. We hypothesise that (1) adults who grew up with a PWMI will experience more PTG as compared to adults who did not. Further, (2) we examine how PTG changes as a factor of participants having a mental illness themselves. On the one hand, it can be expected that increased number of traumatic events (i.e., parent’s plus own mental illness) will lead to more PTG (Fausor et al., 2022). On the other hand, it might be that having a mental illness reduces successful coping by exhausting an individual’s social, cognitive, and emotional resources. Given mixed research on this point, we left the examination of this hypothesis as exploratory.

## Method

### Participants

Online survey data was collected from 162 participants, who were assigned to either the PWMI or the NO-PWMI group according to a set of screening criteria asking about their parents’ mental health status when they were children; the criteria are detailed below (see Design and Materials section). The participants in the PWMI group were further asked whether they currently or previously had a mental illness themselves, and were therefore assigned to either the PWMI-MI or the PWMI-NO-MI group. Out of the data that were collected, 16 were removed due to not meeting the inclusion screening criteria. As a result, data from 146 participants was analysed (18 males, 128 females, *M* age = 32.19 years, *SD* = 13.04, age range = 18–77): *N* = 71 participants were in the PWMI group (*N* = PWMI-MI, *N* = PWMI-NO-MI), and *N* = 75 participants were in the NO-PWMI group. Of the 71 with a PWMI, *N* = 38 participants were in the PWMI-MI group, and *N* = 33 participants were in the PWMI-NO-MI group. Ethical approval was granted by Nottingham Trent University.

A power analysis was conducted to calculate the number of participants needed. Based on Ergün et al. (2018), a study of parental mental illness and PTG, a sample size of 122 was calculated for a one-way ANOVA based on a mean difference of 3.5 with a power of 0.7 and 5% error rate. An opportunity and snowball sampling method using social media and the Nottingham Trent University SONA system, which is comprised mostly of university students, was used to recruit participants. All participants had to be over 18 and be able to read and write in English.

### Design & Materials

This experiment used a between-participant design and had two predictor variables: parental mental illness (PWMI vs. no-PWMI) and own mental health status (has vs. does not have a mental illness). For both hypotheses, the outcome variable was the amount of post-traumatic growth (PTG) experienced.

Parental mental illness was measured with a yes/no question asking whether the participant’s parent had a formally diagnosed mental illness while they were growing up. Participants who reported a PWMI were further asked what their parent’s diagnosis was and if there were multiple diagnoses, and they were also asked if their parent had their mental illness for over 6 months. Participants then completed a purpose-built 6-item checklist comprised of common experiences from the literature of parental mental illness (Källquist & Salzmann-Erikson, 2019). These items were: parent being hospitalised due to their mental illness, parent attempting suicide or exhibiting suicidal behaviour or ideation, participant feeling hopeless and unable to cope with their parent’s mental illness, participant experiencing stigma due to parent’s mental illness, participant feeling socially isolated due to their parent’s mental illness, and participant feeling ashamed due to their parent’s mental illness. On average, participants in the PWMI group reported having had 3.69 experiences (range: 1–6).

Participants who did not have a PWMI were asked to think about a highly stressful event in their childhood and explain it in 5 words, which should include the people involved, where it took place and how they felt about it. This was in order to ensure that there was an event that the Post Traumatic Growth Index (Tedeschi & Calhourn, 1996) could be used to measure and something comparable to the experience of having a PWMI. They were then given an adapted version of the 6-item checklist of common experiences that referred to their childhood highly stressful event (e.g., you/someone else was hospitalised, you experienced stigma etc.). Participants in the no-PWMI group reported having had an average of 2.69 experiences (range: 1–6), indicating that the non-PWMI childhood event they recalled was indeed highly, and similarly, stressful to growing up with a PWMI. Participants with a PWMI were then asked about whether they had ever been diagnosed with a mental illness themselves and if they are still being treated/still have the mental illness.

PTG was measured for all participants using the Post-Traumatic Growth Index (Tedeschi & Calhourn, 1996). This well-established, widely used scale has been shown to have good validity, internal consistency (α = 0.90) and reliability (*r* = .71) (Shakespeare-Finch et al., 2013; Tedeschi & Calhourn, 1996). The Post-Traumatic Growth Index has 21 items, each rated on a scale of 0–5 (0: I did not experience this as a result of my experience to 5: I experienced this change to a very great degree in relation to my experience), with a maximum score of 105. It contains 5 subscales: relating to others, new possibilities, personal strengths, spiritual change, and appreciation of life. The scores of all items within a subscale or across all subscales can be added together to give a subscale score or an overall score, respectively. Higher scores indicate more PTG, with scores below 45 typically considered to indicate relatively low level of growth, and scores over 46 are moderate to high level of growth (Mazor et al., 2016).

### Procedure

Participants filled out a survey set up on Qualtrics that took around 10 min to complete. In order of appearance, the survey items comprised of demographic questions, parental mental illness status (yes/no, diagnosis specification, 6-item checklist), participants’ own mental health status, and the Post-Traumatic Growth Index.

### Analysis

To measure PTG, both overall and sub-scale scores (i.e., relating to others, new possibilities, personal strengths, spiritual change, and appreciation of life) were calculated and used as outcome variables in the analyses. Hypothesis 1 was assessed with one-way ANOVA tests comparing the PWMI group to the NO-PWMI group. Hypothesis 2 was assessed with one-way ANOVA tests within the PWMI group to compare those who had a mental illness themselves (PWMI-MI) to those who *did not* have a mental illness themselves (PWMI-NO-MI). Given the uneven distribution of males and females in our sample, we re-ran all analyses excluding male participants. Since all findings remained unchanged in the female-only sample, here, we report the findings from the entire sample including males.

## Results

The descriptive statistics for each group are presented in Table 1. The most common parental illness was depression, and the most common co-occurring mental illnesses were depression and anxiety, with 15 participants having either condition as co-occurring with another condition (see Table 2). Gender (*F*(1,143) = 0.09, *p* = .77, η^2^ = 0.001), age (*F*(1,143) = 1.29, *p* = .26, η^2^ = 0.01) or parents having a single vs. multiple diagnoses (*F*(1,69) = 2.13, *p* = .15, η^2^ = 0.03) did not have a significant effect on overall PTG scores.

**Table 1:** Sample description by age and gender

Participant Group	Age (*M* ± SD)	Gender (F / M)
PWMI	32.01 ± 11.15	65 / 6
NO- PWMI	32.36 ± 14.68	63 / 12
Difference between PWMI and NO-PWMI	*t*(144) = -0.16, *p* = .87	*X*^2^(1) = 1.92, *p* = .17
PWMI-MI	31.08 ± 9.80	34 / 4
PWMI-NO-MI	33.09 ± 12.61	31 / 2
Difference between PWMI-MIand PWMI-NO-MI	*t*(69) = 0.76, *p* = *.45*	*X*^*2*^(1) = 4.55, *p* = .50

**Table 2:** Types of parental mental illnesses reported, in order of number of occurrence

Mental Illness	Number of occurrences (*N* co-occurrence)
Depression	37 (15)
Bipolar Disorder	20 (12)
Schizophrenia	14 (7)
Anxiety Disorder	12 (15)
Schizoaffective Disorder	8 (6)
Anorexia	7 (4)
Substance Use Disorder	6 (4)
Borderline Personality Disorder	4 (3)
Manic Depression	3 (3)
Attention Deficit Hyperactivity Disorder	3 (3)
Major Depressive Disorder	2 (3)
Dissociative Identity Disorder	2 (2)
Obsessive Compulsive Disorder	2 (2)
Other (Panic disorder, Schizoid Personality Disorder, Anti-Social Personality Disorder, Post-Traumatic Stress Disorder, Personality Disorder, Narcissistic Personality Disorder, Paedophilic Disorder, Specific Phobia)	1 (1)

### Effect of Growing Up with a PWMI on PTG

**Fig. 1:**
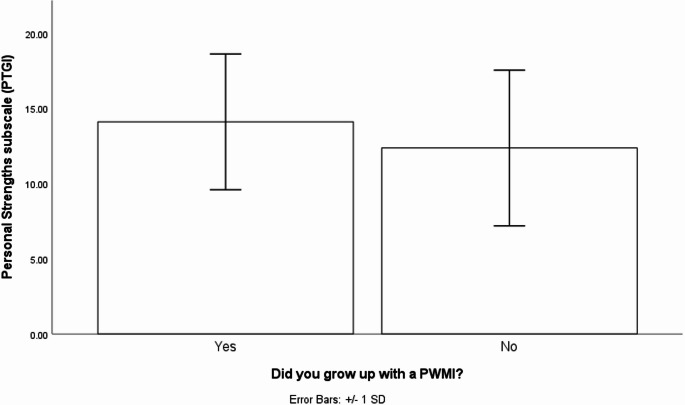
Bar graph showing the means (with +/- 1 standard deviation error bars) of the Personal Strengths subscale for adults who grew up with a PWMI and those who did not

One-way ANOVA tests comparing the PWMI group to the NO-PWMI group showed a significant effect of growing up with a PWMI on personal strengths (*F*(1,114) = 110.34, *p* = .03, η^2^ = 0.03) such that people who grew up with a PWMI (*M*_*PWMI*_ = 14.11, SD_*PWMI*_ = 4.52) had more personal strengths than those who did not grow up with a PWMI (*M*_*No−PWMI*_ = 12.37, SD_*No−PWMI*_ = 5.17, see Fig. 1). The PWMI and NO-PWMI groups did not differ on overall PTG (*M*_*PWMI*_ = 56.63, SD_*PWMI*_ = 18.59, *M*_*No−PWMI*_ = 53.27, SD_*No−PWMI*_ = 21.14, *F*(1,144) = 1.04, *p* = .31, η^2^ = 0.01), relating to others (*M*_*PWMI*_ = 18.18, SD_*PWMI*_ = 7.92, *M*_*No−PWMI*_ = 17.80, SD_*No−PWMI*_ = 9.09, *F*(1,114) = 0.73, *p* = .79, η^2^ = 0.001), new possibilities (*M*_*PWMI*_ = 12.08, SD_*PWMI*_ = 5.99, *M*_*No−PWMI*_ = 11.48, SD_*No−PWMI*_ = 6.07, *F*(1,114) = 0.37, *p* = .55, η^2^ = 0.003), spiritual change (*M*_*PWMI*_ = 2.85, SD_*PWMI*_ = 2.74, *M*_*No−PWMI*_ = 2.76, SD_*No−PWMI*_ = 3.00, *F*(1,114) = 0.31, *p* = .86, η^2^ = 0.00) or appreciation of life (*M*_*PWMI*_ = 9.41, SD_*PWMI*_ = 3.70, *M*_*No−PWMI*_ = 8.85, SD_*No−PWMI*_ = 3.75, *F*(1,114) = 0.810, *p* = .37, η^2^ = 0.01).

### Effect of Having a Mental Illness on PTG

**Fig. 2:**
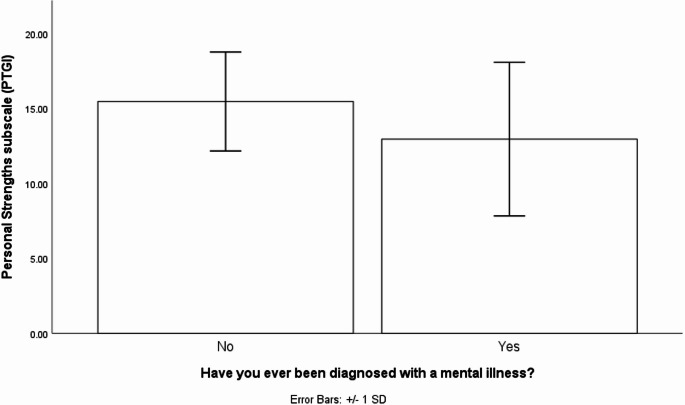
Bar graph showing the means (with +/- 1 standard deviation error bars) of the Personal Strengths subscale for adults with a PWMI who *had* and who *did not have* a mental illness themselves

One-way ANOVA tests in the participants with a PWMI sample comparing those with vs. without a mental illness showed a significant effect of having a mental illness on personal strengths (Welch’s F test due to violation of homogeneity of variance assumption: *F*(1,63.93) = 6.17, *p* = .02, est. ω = 0.07), such that participants without a mental illness (*M* = 14.11, SD = 4.52) had more personal strengths than those that did have a mental illness (*M* = 12.37, SD = 5.17, see Fig. 2). The participants in the PWMI-MI and PRMI-NO-MI groups did not differ on overall PTG (*M*_*No−MI*_ = 59.58, SD_*No−MI*_ = 17.34, *M*_*MI*_ = 54.08, SD_*MI*_ = 19.47, *F*(1,69) = 1.56, *p* = .22, η^2^ = 0.02)., relating to others *(M*_*No−MI*_ = 18.33, SD_*No−MI*_ = 8.70, *M*_*MI*_ = 18.05, SD_*MI*_ = 7.29, *F*(1,69) = 0.78, *p* = .38, η^2^ = 0.01), new possibilities *(M*_*No−MI*_ = 12.76, SD_*No−MI*_ = 5.82, *M*_*MI*_ = 11.50., SD_*MI*_ = 6.16, F(1,69) = 0.75, *p* = .39, η2 = 0.01, spiritual change (*M*_*No−MI*_ = 3.21, SD_*No−MI*_ = 2.88, *M*_*MI*_ = 2.52, SD_*MI*_ = 2.61, *F*(1,69) = 1.11, *p* = .30, η^2^ = 0.02) or appreciation of life *(M*_*No−MI*_ = 9.82, SD_*No−MI*_ = 4.03, *M*_*MI*_ = 9.05, SD_*MI*_ = 3.40, *F*(1,69) = 0.75, *p* = .39, η^2^ = 0.01).

## Discussion

This study examined the extent to which adults who grew up with a PWMI experienced PTG compared to adults who did not, and whether this differed based on the participants’ own mental health status. Supporting Hypothesis 1, we found that participants who had a PWMI had significantly higher personal strengths as compared to participants who did not have a PWMI, though other dimensions of PTG or overall PTG were not different between the two groups. Moreover, we found that among participants with a PWMI, those who didn’t have a mental illness themselves had higher personal strengths as compared to participants who had a mental illness. Personal strengths are associated with a greater ability to handle difficulties, increased feeling of self-reliance, and better acceptance of life outcomes and as well as developing greater mental strength (Tedeschi & Calhoun, 1996). Again, no other dimensions of PTG or overall PTG were different between participants with and without a mental illness.

PWMI not influencing overall PTG is in line with the findings of Ergün et al. (2018). Adults who grew up with a PWMI may not experience significantly higher levels of PTG because of the traumatic nature of having a PWMI and the negative long-term effects associated with it (Yamamoto & Keogh, 2018). PTG is considered to be developed through the reduction of emotional distress after a traumatic experience, which helps the individual to transform their harmful ruminations into purposeful reflection (Mazor et al., 2016). If the individual is still highly distressed about their experience, they may not develop PTG. Consequently, it is important to consider that when trauma is ongoing or continuous, each new event will add to the individual’s fear and anxiety that the traumatic experiences will continue to happen (Afana et al., 2010). Therefore, the trauma cannot be seen to be over and since PTG is growth *post* the trauma, PTG may be difficult to develop if the trauma is ongoing.

Our finding that adults who grew up with a PWMI gained personal strengths from their experiences is consistent with previous literature (Kinsella et al., 1996; Metz & Jungbauer, 2021; Van Parys et al., 2015). The personal strengths subscale measures self-reliance, being able to handle difficulties and ability to accept the way situations work out. Kinsella et al. (1996), used qualitative data to find adults who grew up with a PWMI experienced all these aspects. Growing up with a PWMI can help develop self-reliance and ability to handle difficulties through experiencing parentification, and having to handle “adult-like” situations successfully. Developing greater self-reliance could lead to the development of ‘coping strategies’ to help manage stressful situations and increase resourcefulness. Building a strong sense of self-reliance at a young age could also mean that people who grew up with a PWMI might feel more resilient and able to proactively overcome obstacles in adulthood that their peers may struggle with. For instance, Patrick et al. (2020) found that adults who grew up with a PWMI used reflective practices to make meaning from their adverse experiences which is a vital component in developing PTG (Xu et al., 2016). By developing the ability to accept the adversity they had experienced due to having a PWMI, they were able to make meaning from their experiences and recognise the positives and utilise the personal strengths they had gained.

We further found that participants who did not have a mental illness experienced higher levels of PTG than those who had a mental illness. Individuals with a mental illness may have negative cognitive biases, leading to a decreased ability to cognitively reappraise a negative or difficult situation (Gross et al., 2019). Given that PTG requires engaging in positive meaning-making following a traumatic event, if an individual has a mental illness and is distressed, they may not be able to make a positive meaning and consequently struggle to develop PTG (Xu et al., 2016). Thus, our findings show that while having a PWMI is linked with increased personal strengths, the individual having a mental illness on top of this may incur additional burden that reduces PTG.

We did not find any effects of growing up with a PWMI or participants having a mental illness themselves on other sub-scales of PTG or overall PTG scores. One contributing factor to this could be that adults who grew up with a PWMI can struggle to have relationships or relate to people who did not have a PWMI which may explain why they did not score significantly higher on the relating to others subscale (Källquist & Salzmann-Erikson, 2019). Spiritual change is commonly the lowest scored subscale, which might be because it only has two items making it psychometrically weak (Jordan et al., 2019). The appreciation of life subscale may have not been significant, as both growing up with a PWMI and having a mental illness are continuous experiences. A study examining continuous, rather than one-off, trauma found significant negative correlations with PTG, indicating that it is harder for participants to appreciate their life when the traumatic event is ongoing (Afana et al., 2010; Kira et al., 2013). The new possibilities subscale relates to developing new interests and self-improvement, which may not have been a relevant for adults who grew up with a PWMI and/or those who have a mental illness as caring duties and instability of life circumstances may have prevented these individuals from seeking new opportunities (Trondsen, 2012).

This study compared the participants in the PWMI group to those who did not have a PWMI, but nevertheless experienced a significant adverse childhood experience. In other PTG studies, participants have often been matched by age, education level, and in a breast cancer study, the length of time since cancer diagnosis was used to compare PTG in this time period (Cordova et al., 2001). Our more conservatively defined no-PWMI comparison group enabled us to specifically examine the PTG resulting from growing up with a PWMI, rather than from experiencing any negative childhood event. This might partially explain the non-significant differences between the PWMI and no-PWMI groups, as both groups reported high levels of PTG (i.e., over the typical cut-off of 45). Undoubtedly there are differences between an ongoing stressor such as growing up with a PWMI compared to a single, possibly time-limited, stressful event. For instance, in the case of growing up with a PWMI, there is the possibility for the development of complex trauma due to experiencing multiple pervasive traumatic events. Nevertheless, the impact of single traumatic events during childhood should not be underestimated as being less impactful on a person’s development into adulthood (Lewis et al., 2021). Crucially, for the purposes of this study, both groups will have had to reflect and make meaning from their experiences in order to develop PTG. To further validate that both groups had trauma-like negative experiences, we developed a 6-item checklist based on existing literature (Källquist & Salzmann-Erikson, 2019). Future studies can use this checklist as eligibility criteria, though caution should be paid to not conflate this with trauma severity and the self-reported nature of the experiences.

A major limitation of this study is its cross-sectional nature, preventing us from delineating whether it is the childhood PWMI experiences that led to the observed PTG or other knock-on experiences that might be related to it. Future research should examine this question using a longitudinal design and further group comparisons and could use qualitative data to provide further insight into the qualitative nature of PTG. Another direction for future research is to examine gender differences in PWMI and PTG experiences as our sample comprised mostly of women, a recruitment challenge which limits the generalisability of the results also encountered in previous studies looking at parental mental illness (Metz & Jungbauer, 2021; Murphy et al., 2018). The sample itself may have introduced bias into the results due to recruitment being mostly completed through snowball sampling. Another limitation of this study is that the sample only includes with an official diagnosis to ensure reliability of cases in an online survey method. Given missed and delayed diagnoses in mental illnesses (Mansour et al., 2020), this approach may have miss some people, and future research should look into a broader range of mental health issues.

The implications of these findings could be useful for the implementation of programmes such as Child Talks, which is an intervention targeted at individuals 0–25 years old who have a PWMI (Kristensen et al., 2022; Reedtz et al., 2012). It delivers psychoeducation to parents who have a mental illness and their children. It allows the child of the PWMI to ask questions and share their concerns. Although there have been no studies conducted on the effectiveness of the intervention, it hopes to strengthen the parent’s and child’s knowledge about the parent’s mental illness. It is hoped that by allowing room for discussion, the intervention will strengthen the child’s social and emotional support as well as their ability to successfully cope with their parent’s mental illness. It can also be used to detect if the child themselves may have a mental illness. The findings of this study, in relation to the findings of personal strengths, could be used to help support this intervention by helping the children identify and develop their personal strengths to help them better cope with their parent’s mental illness. Individual interventions may also be beneficial for people with a PWMI so they can discuss their feelings in a space without fear of upsetting their parent and help them make sense and meaning of their experiences. Using a strengths-based approach could help the individual to recognise their inherent strengths such as resilience and empathy. Having a space to discuss their experiences may also help the individual to gain a sense of community and encourage help-seeking (Jetten et al., 2012), which until then the individual may have struggled with due to stigma around mental illness and being afraid of judgement. Through the use of interventions aspects of PTG can be promoted to help individuals view the event/s differently.

In conclusion, while having a PWMI can be undeniably traumatic and cause psychological distress, it can also have positive effects. This study, examining PTG in adults who had a PWMI and a mental illness themselves, provides evidence that adults who had a PWMI experienced significantly higher levels of personal strengths, especially if they themselves did not have a mental illness. Through the application of PTG theory these findings highlight the potential for building personal growth and resilience amidst adverse life experiences insofar as individuals have the resources and capacity reflect positively on their experiences. From the distressing experiences associated with having a PWMI can come the possibility for positive growth and improved personal strengths.
